# BH3 Mimetics in Hematologic Malignancies

**DOI:** 10.3390/ijms221810157

**Published:** 2021-09-21

**Authors:** Pavel Klener, Dana Sovilj, Nicol Renesova, Ladislav Andera

**Affiliations:** 1 First Department of Internal Medicine-Hematology, General University Hospital in Prague, 121 08 Prague, Czech Republic; 2 First Faculty of Medicine, Institute of Pathological Physiology, Charles University, 121 08 Prague, Czech Republic; nicol.renesova@lf1.cuni.cz; 3 Institute of Biotechnology CAS/BIOCEV, 252 50 Vestec, Czech Republic; dana.sovilj@ibt.cas.cz (D.S.); Ladislav.Andera@ibt.cas.cz (L.A.); 4 Institute of Molecular Genetics CAS, 142 20 Prague, Czech Republic

**Keywords:** apoptosis, BH3 mimetics, venetoclax, hematologic malignancies, targeted therapy, resistance, biomarkers

## Abstract

Hematologic malignancies (HM) comprise diverse cancers of lymphoid and myeloid origin, including lymphomas (approx. 40%), chronic lymphocytic leukemia (CLL, approx. 15%), multiple myeloma (MM, approx. 15%), acute myeloid leukemia (AML, approx. 10%), and many other diseases. Despite considerable improvement in treatment options and survival parameters in the new millennium, many patients with HM still develop chemotherapy-refractory diseases and require re-treatment. Because frontline therapies for the majority of HM (except for CLL) are still largely based on classical cytostatics, the relapses are often associated with defects in DNA damage response (DDR) pathways and anti-apoptotic blocks exemplified, respectively, by mutations or deletion of the *TP53* tumor suppressor, and overexpression of anti-apoptotic proteins of the B-cell lymphoma 2 (BCL2) family. BCL2 homology 3 (BH3) mimetics represent a novel class of pro-apoptotic anti-cancer agents with a unique mode of action—direct targeting of mitochondria independently of *TP53* gene aberrations. Consequently, BH3 mimetics can effectively eliminate even non-dividing malignant cells with adverse molecular cytogenetic alterations. Venetoclax, the nanomolar inhibitor of BCL2 anti-apoptotic protein has been approved for the therapy of CLL and AML. Numerous venetoclax-based combinatorial treatment regimens, next-generation BCL2 inhibitors, and myeloid cell leukemia 1 (MCL1) protein inhibitors, which are another class of BH3 mimetics with promising preclinical results, are currently being tested in several clinical trials in patients with diverse HM. These pivotal trials will soon answer critical questions and concerns about these innovative agents regarding not only their anti-tumor efficacy but also potential side effects, recommended dosages, and the optimal length of therapy as well as identification of reliable biomarkers of sensitivity or resistance. Effective harnessing of the full therapeutic potential of BH3 mimetics is a critical mission as it may directly translate into better management of the aggressive forms of HM and could lead to significantly improved survival parameters and quality of life in patients with urgent medical needs.

## 1. Introduction

Caspase-dependent apoptosis represents the most flexible and widely used regulated cell death (RCD) modality utilized in the animal kingdom by both invertebrates and vertebrates during embryonic development as well as in adult organisms with rapid and effective efferocytosis of dead cells [1]. Apoptotic signaling from a simplified mode (e.g., in C. elegans) evolved into a vast, though tightly regulated, signaling network in mammals. The intermediate as well as effector proteins in mammalian apoptotic signaling are specific proteases—caspases—which are both mediators of upstream apoptotic signaling (initiator caspases activated in specialized multiprotein complexes) and executioners of this signaling (effector caspases activated by active initiator caspases as well as by certain other proteases) [2,3]. Apoptotic signaling in mammalian cells can be triggered by exogenous/extrinsic stimuli such as death/dependence receptors or cytotoxic perforin/granzymes-containing granules and by a range of intrinsic stimuli. The intrinsic triggers of apoptotic signaling are associated with various cellular stresses such as damaged DNA, proteins, lipids (also ferroptotic signaling), organelles, increased levels of reactive oxygen species (ROS), energo-metabolic perturbations, and more. Importantly, cells with mitochondria primed by death signals have been shown to be addicted to anti-apoptotic BCL2 molecules (primed for death), which can be exploited therapeutically [4]. The majority of current anti-cancer drugs represent effective indirect activators of intrinsic mitochondria-dependent apoptotic signaling. The efficient triggering of either apoptotic signaling pathway relies on pronounced activation of the initiator caspases-8 and/or -9 in respective multiprotein complexes—the death-inducing signaling complex (caspase-8) and apoptosome (caspase-9) [2]. Subsequent activation of a cohort of effector caspases (caspase-3, -6, and -7) underlines the destructive phase of apoptotic signaling with hundreds of cleaved cellular proteins. Caspase-dependent apoptosis also represents the predominant non-inflammatory RCD mode triggered in the majority of hematologic and other malignancies [5]. Apoptotic blocks that prevent cancer cells from initiating programmed cell death in response to various pro-apoptotic stimuli represents one of the recognized hallmarks of cancer [6,7].

## 2. BCL2 Family of Apoptosis Regulators

**Figure 1:**
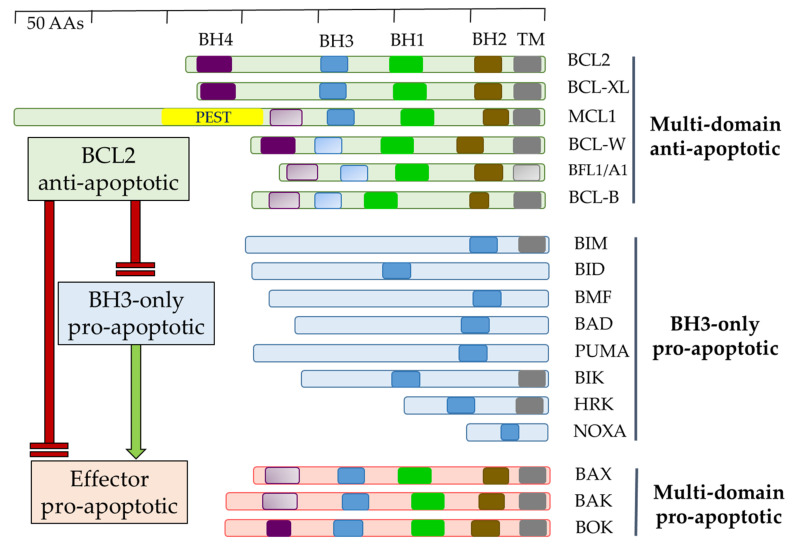
Overview of mammalian proteins of the BCL2 family. Multidomain anti-apoptotic and pro-apoptotic BCL2-homology (BH) BH1-BH4 domains, and transmembrane (TM) domains are shown as opaque; presumed BH and TM domains are shown as transparent. PEST—signal peptide for protein degradation.

The BCL2 family of proteins contains 1–4 evolutionary-conserved BCL2 homology (BH) domains and can, in respect to cell survival, be formally divided into two groups—a/inducing/enhancing or b/suppressing intrinsic/mitochondria-linked apoptosis. The former can be further subdivided into two groups—multidomain effectors of the intrinsic apoptosis BAX, BAK1, and BOK and BH3-only sentinels/activators/, which transduce a pro-apoptotic stress signal to the multidomain BCL2 family proteins (Figure 1) [8].

The anti-apoptotic BCL2 family comprises six multi-BH domain (BH1–BH4) proteins, namely BCL2, BCL-XL, MCL1, BCL-W, BFL-1/A1, and BCL-B, which maintain balance by restricting multidomain pro-apoptotic proteins and sequestering BH3-only sentinels. BCL-XL and MCL1 are essential for embryonic development as mice with eliminated expression of either one die during embryogenesis (day 13 or during preimplantation) (Table 1).

BH3-only pro-apoptotic BCL2 proteins form a diverse group of molecules that are induced/activated in response to various cellular stress signals. They can be released from cellular compartments (e.g., BCL2 interacting protein [BIM] or BCL2 modifying factor [BMF] from the cytoskeleton), transcriptionally upregulated (e.g., PUMA and NOXA in response to DNA damage via the *TP53*-dependent pathway), or activated (e.g., BH3 interacting domain death agonist [BID] cleaved by caspase-8 into truncated (t)BID) [9]. Stress stimuli that BH3-only proteins can recognize as apoptotic are heterogeneous in nature and include cytokine deprivation, anoikis, antigen receptor signaling, activation of oncogenes, DNA damage, chemotherapy drugs, or irradiation with UV light or γ-rays (Willis SN and Adams JM, 2005) [10]. Multidomain BCL2 family proteins contain, in addition to BH domains, a C-terminal transmembrane domain (TM) allowing them to associate with intracellular membranes such as the mitochondrial outer membrane (MOM), endoplasmic reticulum (ER), and nuclear membranes [11]. Their embedding into MOM is instrumental both for their anti-apoptotic and pro-apoptotic roles. During cellular homeostasis anti-apoptotic BCL2 proteins restrict BCL2 associated X (BAX) or BCL2 antagonist/killer 1 (BAK1) from being multimerized by direct binding to them as well as buffering basal levels of BH3-only proteins. Upon pro-apoptotic cellular stress, induction/activation of various BH3-only BCL2 proteins leads to the release of BAX/BAK1 effector proteins from the anti-apoptotic BCL2 molecules. Released BAX/BAK1 then unfold in the MOM and, with the assistance of some BH3-only proteins (e.g., BID, BIM), or rather their C-terminal parts [12,13], they initially form homodimers gradually aggregating in the MOM and forming channels allowing the release of cytochrome c and other proteins from the mitochondrial intermembrane space in a process called mitochondrial outer membrane permeabilization (MOMP) [14,15]. Interestingly, MOMP does not always lead to irreversible apoptosis, but a small fraction of mitochondria with permeabilized MOM in the cell can be tolerated [16]. Consequently, MOMP no longer strictly defines the point of no return in the apoptotic process, but this “minority MOMP” can promote genome instability and contribute to the complex process of tumorigenesis.

## 3. BCL2

### 3.1. BCL2 and Its Role in the Regulation of Apoptosis and Other Cellular Processes

BCL2, a 25 kD protein coded on 18q21.3, was originally discovered as a translocation partner in patients with follicular lymphoma (FL) and other B-cell malignancies [17,18,19]. It was soon recognized that BCL2 was involved in the regulation of apoptosis and that it cooperated with the *MYC* oncogene in lymphoma pathogenesis [20,21,22,23]. BCL2 contains a conserved globular BCL2 core comprising eight α-helices and a C-terminal transmembrane helix [24]. The C-terminus contains hydrophobic amino acids, which allow posttranslational localization of the BCL2 protein into intracellular membranes, predominantly the MOM, endoplasmic reticulum, and nuclear envelope [25]. Studies on BCL2 knockout mice revealed post-natal growth retardation, development of polycystic kidneys, and a massive loss of lymphocytes, probably due to involution of the spleen [26,27]. Interestingly, loss of even one allele of the pro-apoptotic BH3-only protein BIM rescued all of these abnormalities, pointing to an essential role of BCL2-BIM interactions [28]. In addition to BIM, other BH3-only proteins, BAD, PUMA, and BID, interact with BCL2 upon diverse apoptotic stimuli and sequester it from the pro-apoptotic BAX or BAK1 [10]. Besides their undisputed role in apoptosis, BCL2 and other anti-apoptotic BCL2 family members participate in other cellular processes [29]. Moreover, BCL2 negatively regulates autophagy by direct binding to Beclin-1. BCL2 also participates in the regulation of cellular calcium levels [30] via its interaction with ligand-gated Ca^2+^ channels in the ER and might serve as a link between the ER and mitochondria [31,32]. Together with BCL-XL_,_ BCL2 can impose cell cycle delay by prolonging the G_0_ phase through upregulation of the cell cycle inhibitor p27 [33,34]. In addition to its crucial apoptosis-modulating role, BCL2 enhances oxidative phosphorylation through binding to cytochrome c oxidase [35,36].

### 3.2. BCL2 and Hematologic Malignancies

In addition to follicular lymphoma (FL), overexpression of BCL2 through several distinct molecular mechanisms was found in most B-cell lymphoproliferative disorders and myeloid malignancies (Table 2) [37,38,39,40,41,42,43,44,45,46,47,48,49,50,51,52]. BCL2 gene aberrations and/or upregulated BCL2 protein repeatedly correlated with shorter PFS or OS [53,54,55].

### 3.3. BCL2 as a Therapeutic Target and a BH3 Mimetics Class of Agents

**Figure 2:**
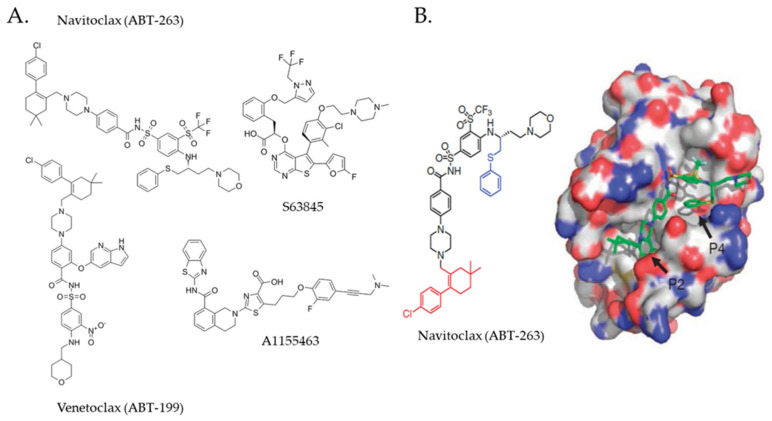
Selected BH3 mimetics targeting the anti-apoptotic BCL2 family proteins. (**A**) Molecular formulas of representative BH3 mimetics binding to/blocking BCL2/BCL-XL (navitoclax), BCL2 (venetoclax), MCL1 (S63845), or BCL-XL (A1155463); (**B**) Graphical representation of navitoclax and its binding to the hydrophobic groove of BCL2 protein (P2, P4—navitoclax-binding BCL2 hydrophobic pockets). Reproduced from [65] with permission from Springer Nature publishing house.

Since the discovery of its role in malignant cell survival, BCL2 has appeared as a rational therapeutic target, but the early attempts to inhibit BCL2 were rather disappointing. An antisense oligonucleotide oblimersen (G3139) did not demonstrate survival benefit in patients with acute myeloid leukemia (AML) and multiple myeloma (MM) [56,57]. Similarly, obatoclax, the first BH3 mimetic tested in human subjects with relapsed/refractory (R/R) CLL had only limited clinical activity [58]. BH3 mimetics represent small molecule chemicals designed to bind and block the prosurvival groove of anti-apoptotic BCL2 family proteins thereby eliciting dose-dependent activation of BAX and BAK1 [59]. Another BH3 mimetic, a dual BCL2/BCL2L1 (BCL-XL) inhibitor navitoclax (ABT-737) demonstrated clinical activity in patients with R/R CLL, but the overall response rate was rather low and administration of navitoclax was associated with severe “on-target” thrombocytopenia attributable to high-affinity inhibition of BCL-XL in megakaryocytes [60,61,62]. ABT-737, a predecessor of orally applicable ABT-263 was prepared by nuclear magnetic resonance (NMR)-assisted structure-based modulation of a compound selected from a chemical library that strongly interacted with the BH3-binding hydrophobic groove of BCL-XL/BCL2 and induced apoptosis of various human cancer cell lines at submicromolar concentration (Figure 2) [63]. A similar strategy was used for the design of the first nanomolar inhibitor of BCL2 ABT-199/venetoclax for the generation of the first selective inhibitor of MCL1 S63845, as well as for the derivation of highly-specific BCL-XL inhibitors A1155463 and WEHI-539 [64,65,66,67,68]. Thus, at least for BH3 mimetics, structure-based design and fine modulation of their selectivity and activity undoubtedly represent a perspective approach for enlarging the family of potent and safe anti-cancer drugs targeting anti-apoptotic BCL2 family proteins [69,70].

### 3.4. Venetoclax

In sharp contrast, the nanomolar BCL2-specific inhibitor venetoclax (ABT-199, GDC-0199) proved effective in most hematologic malignancies and was soon approved for the therapy of patients with CLL and AML (Table 3) [65,71,72,73,74,75]. Besides the currently approved indications, venetoclax demonstrated promising clinical activity in patients with mantle cell lymphoma (MCL), a lymphoma subtype molecularly characterized by the translocation t(11;14) leading to overexpression of cyclin D1 [76]. Notably, the most robust in vitro and in vivo activity of venetoclax in MM was also observed in the cell lines and subcohort of patients harboring this translocation. Similarly, venetoclax can induce deep remissions in patients with t(11;14)-positive AL amyloidosis [77]. Despite the clear positive correlation between t(11;14) and sensitivity to venetoclax, the precise molecular rationale for the observed BCL2 dependence of the cases with t(11;14) remain largely elusive [78]. In treatment-naive diffuse large B-cell lymphoma (DLBCL) patients, venetoclax, in combination with standard immunochemotherapy (R-CHOP), improved PFS, especially in the BCL2-positive (i.e., detectable by immunohistochemistry (IHC)) subgroup [79,80].

#### 3.4.1. Venetoclax Mode of Action and Tumor Lysis Syndrome

Unlike chemotherapy, venetoclax can trigger cell death not only in the actively dividing cells but also in the resting malignant cells, including cells with deregulated DNA damage response pathways, namely aberrations of tumor suppressor genes *TP53*, *ATM*, and complex karyotype changes [81,82]. Indeed, venetoclax can induce deep remissions, including molecular remissions, as measured by minimal residual disease (MRD) assessment in CLL and AML patients [83,84]. On the other side, the massive activation of apoptosis in cancer cells can lead to serious side effects including the life-threatening tumor lysis syndrome (TLS), especially in patients with a high tumor burden, heavily leukemized cases, or patients with renal impairment [85,86,87]. In some studies, venetoclax is being studied as a consolidation after chemotherapy-based debulking for targeted MRD eradication (with no risk of TLS) [82].

#### 3.4.2. TP53 Mutational Status and Venetoclax Efficacy

Despite the efficacy of venetoclax toward *TP53*-deleted/mutated malignant cells has been repeatedly confirmed by many independent researchers, the most recent data brought evidence that the tumor suppressor function of p53 is nevertheless requisite for sustaining durable responses to venetoclax [88,89]. A subcohort of AML patients with mutated *TP53* treated with the combination of venetoclax and decitabine had significantly lower response rates and shorter survival parameters compared to patients with wild-type *TP53* [90].

#### 3.4.3. Non-Canonical Modes of Venetoclax Action

Besides blocking prosurvival BCL2 signaling in various types of malignant cells, venetoclax shows some non-canonical anti-tumor activities. It has been shown to directly suppress oxidative phosphorylation (OxPhos) in leukemia stem cells leading to their targeted elimination [91]. This anti-metabolic activity of venetoclax was synthetically lethal with inhibitors of respiratory complex I [92]. Notably, venetoclax-induced metabolic reprogramming was found to be independent of BCL2 expression [93]. This off-target metabolic effect could be responsible for some of the observed venetoclax side effects and lead to unexpected drug–drug interactions. Additionally, venetoclax directly enhances T cell-mediated cytotoxicity by increasing reactive oxygen species generation through the inhibition of respiratory chain supercomplexes in non-malignant T cells [94]. Interestingly, metabolic reprogramming conferred acquired venetoclax resistance in lymphoid malignancies [95].

#### 3.4.4. Mechanisms of Resistance to Venetoclax

**Figure 3:**
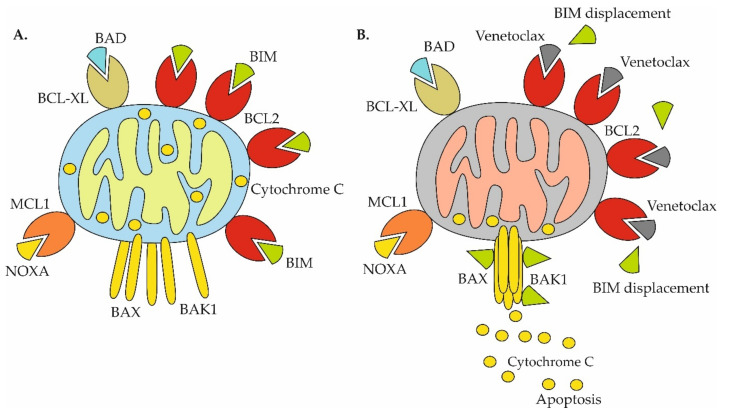
Mechanisms of venetoclax-triggered apoptosis in cells primed for death. (**A**) Cells primed for death: anti-apoptotic BCL2 proteins (BCL2, BCL-XL, MCL1) are fully occupied by pro-apoptotic BH3-only proteins (BIM, BAD, NOXA); (**B**) Venetoclax causes displacement of BIM from BCL2 with subsequent assembly of BAX/BAK1 multiprotein complex, mitochondrial outer membrane permeabilization and release of cytochrome c and activation of apoptosis.

Venetoclax-induced release of BIM sequestered by BCL2 represents the dominant mode of action of the BCL2 inhibitor (Figure 3A,B).

**Figure 4:**
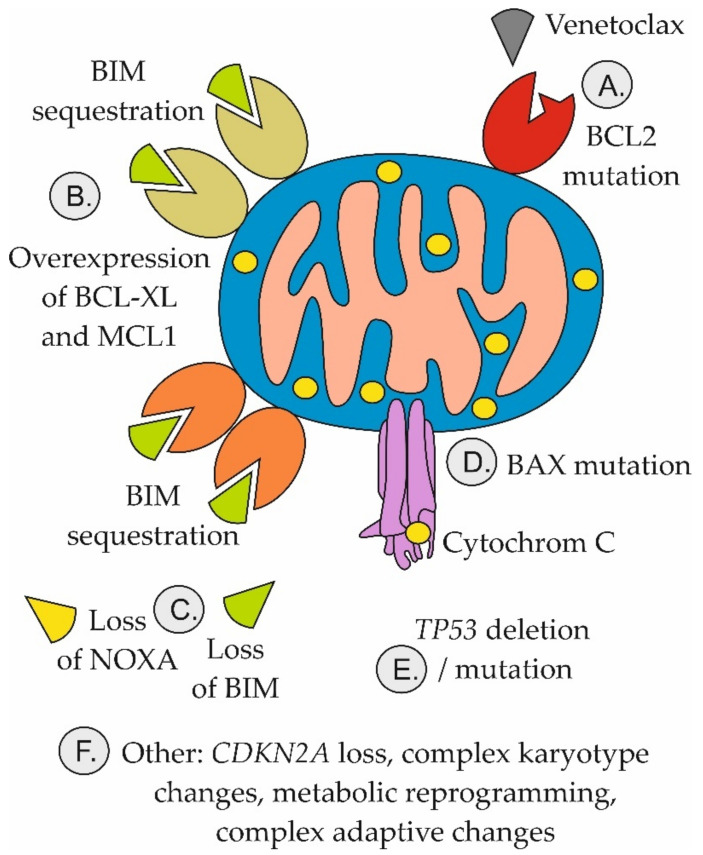
Mechanisms of resistance to venetoclax. (**A**) Mutation of BCL2 prevents binding of venetoclax to BCL2; (**B**) Adaptive overexpression of BCL-XL and/or MCL1 leads to sequestration of BIM released from BCL2; (**C**) Loss of NOXA or BIM attenuates the potential of venetoclax to trigger apoptosis; (**D**) BAX mutations prevent activation of mitochondrial apoptosis; (**E**) TP53 aberrations block mitochondrial apoptosis and facilitate selection of venetoclax resistance clones; (**F**) Other mechanisms include multiple genetic, epigenetic, and metabolic changes, the role of which in mediating venetoclax resistance are only partially understood.

Similarly, upregulation of other key anti-apoptotic proteins, MCL1 and/or BCL-XL, was repeatedly associated with acquired resistance to venetoclax (Figure 4).

Reduced mitochondrial priming was confirmed in AML primary cells and patient-derived xenograft (PDX) models [96]. Detailed molecular mechanisms that underlie the observed upregulation of MCL1 and BCL-XL remain only partially understood. First, NFκB-mediated upregulation of BCL-XL because of CD40-CD154 ligation was repeatedly associated with resistance to venetoclax in CLL cells in the lymph node microenvironment [97]. In addition to that, BCL-XL is directly transactivated by hypoxia-inducible factor 1-alpha (HIF1-alpha) [98,99]. It can be speculated that leukemia or lymphoma cells that reside in the hypoxic lymph node microenvironment might be selected for venetoclax resistance through multiple adaptive steps in a Darwinian fashion. Recently, Haselager et al. demonstrated that BCL-XL appears to play a more important role in mediating resistance to venetoclax than MCL1 [100].

*BCL2* mutations that reduce venetoclax binding sufficient to confer resistance, e.g., *BCL2* Gly101Val, have been reported in CLL patients [95,101,102,103]. Importantly, cells that did not harbor *BCL2* mutations had overexpressed BCL-XL. The data clearly demonstrate a polyclonal nature of the acquired venetoclax resistance. In contrast to CLL, *BCL2* mutations are largely dispensable for acquired venetoclax resistance in AML [104]. Instead, somatic mutations of *PTPN11* and *KRAS*, and upregulation of *BCL2BFL-1/A1*, *CLC7A*, and *SF3B1* conferred resistance to venetoclax [105]. Likewise, the absence of BAX or NOXA (PMAIP1), overexpression of BCL-XL or MCL1, and mutation of *TP53* all impart venetoclax resistance [106,107]. Other mechanisms of venetoclax resistance in AML include disrupted mitochondrial homeostasis with increased OxPhos as a result of upregulated amino acid and/or fatty acid oxidation [108,109]. In MCL, venetoclax resistance is also predominantly associated with non-BCL2 gene alterations including *TP53*, *CDKN2A*, *KMT2C/D*, *SMARCA4*, or *NOTCH2* [110].

#### 3.4.5. Rational Venetoclax Combinations

**Figure 5:**
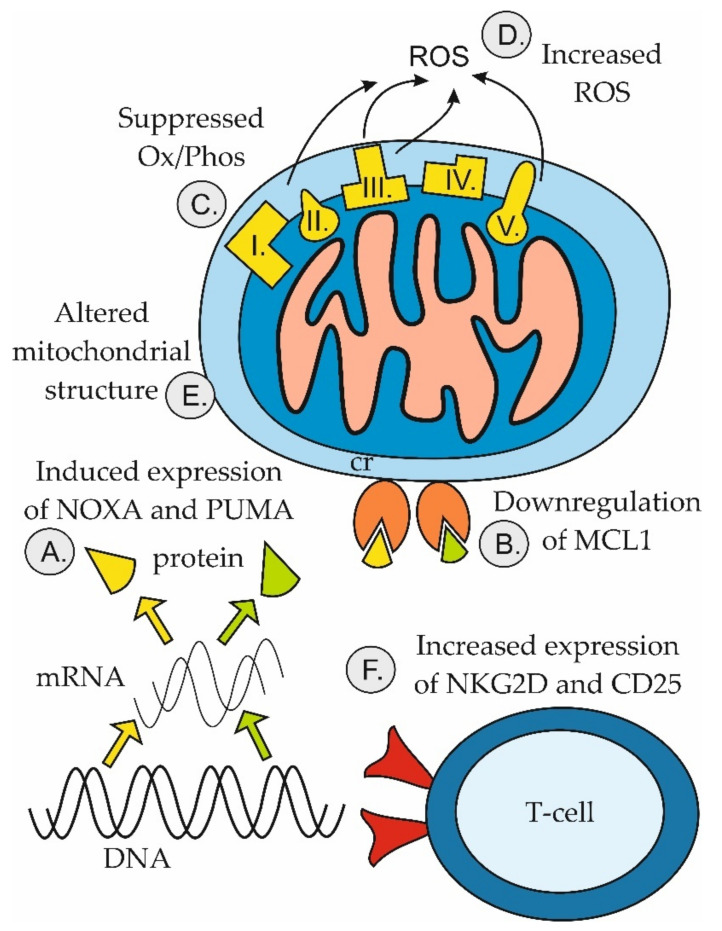
Mechanisms of anti-leukemic synergy between venetoclax and hypomethylating agents. (**A**) HMA-induced expression of pro-apoptotic genes *PMAIP1* (coding for NOXA, yellow) and *PUMA* (green); (**B**) HMA-induced downregulation of anti-apoptotic MCL1 protein; (**C**) Venetoclax-induced suppression of oxidative phosphorylation (Ox/Phos); (**D**) Venetoclax-mediated increase in production of reactive oxygen species (ROS); (**E**) Venetoclax-induced alteration of mitochondrial structure; (**F**) Venetoclax-mediated increase in expression of CD25 and NKG2D on T cells [107,111,112,113].

Venetoclax in combination with hypomethylating agents (HMA, azacytidine, and decitabine) or with anti-CD20 monoclonal antibodies (rituximab, obinutuzumab) belong to the only currently approved therapeutic combinations in human subjects (Table 4). Molecular mechanisms responsible for the observed synthetic lethality between venetoclax and HMA are summarized in Figure 5.

Given its anti-tumor mode of action and well-documented single-agent activity across diverse hematologic malignancies, venetoclax is currently being tested in numerous clinical trials in combination with a wide range of targeted agents and conventional cytostatics (Table 4).

Co-targeting BCL2 and MCL1 anti-apoptotic proteins with different BH3 mimetics has shown promise in numerous preclinical studies and is currently being evaluated both preclinically and in several clinical trials [114]. Venetoclax in combination with a Bruton tyrosine kinase inhibitor (ibrutinib, acalabrutinib), with or without an anti-CD20 antibody (rituximab, obinutuzumab) represent other promising innovative (chemotherapy-free) regimens for diverse B-cell malignancies (CLL, MCL, and Waldenström macroglobulinemia) (Table 4) [115,116]. Of note, it was demonstrated that ibrutinib and venetoclax target distinct compartments of CLL cells [117]. Such a compartmental synergy represents another form of synthetic lethality, which does not augment anti-tumor efficacy mechanistically within a single malignant cell but which targets different subpopulations of cancer cells residing in specific microenvironmental niches and protected by distinct apoptotic blocks. As demonstrated in the combination of venetoclax and HMA, the synergistic effect may also be mediated by non-malignant cells of the tumor microenvironment, e.g., T-lymphocytes (Figure 5). This phenomenon, however, may be difficult or even impossible to model in vitro using established cell lines, but calls for more complex in vivo models or ex vivo analyses.

#### 3.4.6. Next-Generation BCL2 Inhibitors

Several next-generation BCL2 inhibitors are currently being evaluated in several phase 1 clinical trials assessing their safety and the recommended phase 2 doses (Table 5). S655487 (VOB560) and BGB-11417 are potent and highly selective BCL2 inhibitors with putative activity toward *BCL2* mutations (e.g., G101V). APG-2575 demonstrated synthetic lethality with Bruton’s tyrosine kinase (BTK) and mouse double minute 2 homolog (MDM2) inhibitors in preclinical models of DLBCL [118].

## 4. MCL1

### 4.1. MCL1 and Its Role in the Regulation of Apoptosis and Other Cellular Processes

Myeloid cell leukemia 1 (MCL1) was discovered as a prosurvival gene expressed during phorbol ester-induced differentiation of ML-1 human myeloid leukemia cell line [119]. Endogenously, MCL1 is transcribed at various stages of hematopoietic cell differentiation in response to specific growth factors and cytokines and its major role is the promotion of cell viability. Conversely, the loss of MCL1 expression results in programmed cell death, e.g., MYC-driven apoptosis. As in the case of BCL2, MCL1 can be found not only in the outer mitochondrial membrane but also in the endoplasmic reticulum, nuclear envelope, and in the form of a heterodimer with BAX also in the cytosol [120,121]. MCL1 has a similar structure to other anti-apoptotic BCL2 family proteins, with three BH1-3 domains, a putative BH4 domain, and a BH3-binding hydrophobic groove [122]. However, in contrast to the other members of this group, MCL1 contains an extended N-terminal regulatory domain with multiple phosphorylation sites [123,124]. MCL1 is a PEST (P, Pro; E, Glu; S, Ser; T, Thr)-containing short-lived protein and its expression is tightly regulated via ubiquitination by E3 ligases such as MCL1 Ubiquitin Ligase E3 (MULE) and by its interaction with the BH3-only protein NOXA [125,126,127]. In addition to its specific interaction with NOXA, the anti-apoptotic function of MCL1 can be disrupted by other BH-3 only proteins, particularly BIM, BID, and PUMA [10,128]. In contrast to BCL2, MCL1 knockout mice prematurely die during embryogenesis, already at the peri-implantation stage, due to a trophectoderm defect [129]. Conditional, lineage-specific elimination of MCL1 expression during embryogenesis or in the adult mice confirmed a key role of MCL1 in the cardiac homeostasis, the survival of hematopoietic stem cells, lymphocytes, and neural precursors, the development of B-cells, the formation and maintenance of germinal center B-cells, and the development and survival of plasma cells, and naive and memory T cells [130,131,132]. In addition to the anti-apoptotic function of MCL1, its shorter variant, “MCL1 short” (MCL1-S, 36 kD), which is localized in the inner mitochondrial membrane, likely participates in mitochondrial physiology and respiration via the stabilization of respiratory complexes and maintenance of cristae [133]. Moreover, MCL1-S interacts with very long-chain acyl-CoA dehydrogenase modulating its activity and, thus, participating in lipid metabolism [134].

### 4.2. MCL1 and Hematologic Malignancies

It has been demonstrated that diverse solid cancers and hematologic malignancies harbor amplifications of the 1q21 region that codes for MCL1 [135,136]. Additionally, it has also been proven that targeted downregulation of the MCL1 gene in the tumors with 1q21 amplification decreases viability of these tumors. Moreover, while *BCL2* transgenic mice only develop tumors with a low frequency or after cooperation with other oncogenes (e.g., *MYC*), *MCL1* transgenic mice were shown to develop lymphomas (predominantly FL and DLBCL) with high probability (>80%), although with a long latency (over 6 months) [22,137]. The critical role of MCL1 during MYC-driven lymphomagenesis and for the continued survival of lymphoma cells has been repeatedly demonstrated [138]. DLBCL, the most prevalent lymphoma subtype, can be divided into BCL2 and MCL1-dependent categories [46]. MCL1 was reported to be deregulated in a significant fraction of activated B-cells (ABC) DLBCL [139]. Overexpression of MCL1 is a negative prognostic marker in CLL and AML [140,141,142]. Burkitt lymphoma (inherently BCL2-negative) and a subset of BCL2-negative DLBCL are strongly dependent on MCL1 for survival. Multiple myeloma with 1q21 amplification is highly sensitive to MCL1 inhibition [143].

### 4.3. MCL1 Inhibitors

Similarly to BCL2, targeting MCL1 effectively eradicates cancer cells irrespective of their *TP53* mutational status [144]. Several MCL1 inhibitors are currently being evaluated in clinical trials (Table 6). AZD5991 showed potent anti-tumor activity on animal models of AML and multiple myeloma [145]. Another MCL1 inhibitor, AMG-176, was active in CLL patients, and its combination with low-dose venetoclax proved synergistic [146]. However, it must be stressed that MCL1 inhibition has been associated with significant side effects including cardiotoxicity [147]. The safety profile (not efficacy) will probably be the deciding factor in the future trials evaluating the combined inhibition of BCL2 and MCL1.

### 4.4. CDK Inhibitors for Targeted MCL1 Inhibition

As mentioned above, MCL1 is a short-lived anti-apoptotic protein. Consequently, MCL1 inhibition can be effectively achieved not only at the protein level but also transcriptionally. Several CDK inhibitors have been described to effectively suppress mRNA and protein levels of MCL1 [148,149]. Such inhibitors can eliminate MCL1-dependent malignant cells and synergize with venetoclax [148]. CDK7 inhibitor THZ1 was shown to be synthetically lethal with venetoclax in MM through transcriptional downregulation of MCL1, BCL-XL, and MYC [150]. CDK9 inhibitor AZD4573 can overcome intrinsic resistance to venetoclax by inhibiting BCL2BFL-1/A1 in preclinical models of lymphomas [151].

## 5. BCL-XL

### 5.1. BCL-XL and Its Role in Regulation of Apoptosis and Other Cellular Processes

BCL-X, another key regulator of apoptosis, encoded by *BCL2L1* gene, exists in two forms: a long form designated BCL-XL, an anti-apoptotic protein with BH1-4 domains, and a shorter variant called BCL-XS lacking BH1 and BH2 domains [152]. BCL-XL was the first identified BCL2 homolog with the highest sequence and a structural similarity to BCL2 [153]. BCL-XL knockout mice prematurely die during embryogenesis due to extensive apoptosis of immature hematopoietic cells and neurons [154]. BCL-XL has a C-terminal hydrophobic helix with a particular mitochondrial signal sequence, which localizes this protein to MOM [155]. Due to the structural flexibility of its binding groove, BCL-XL has a high affinity to all major BH3-only proteins (BIM, BMF, BAD, BIK, HRK, PUMA, BID) as well as to BAX and BAK1 [156,157]. In addition to binding to and preventing allosteric activation of BAX and BAK1, BCL-XL can shuttle BAX to cytosol, thereby reducing its levels in mitochondria [158]. Similarly to BCL2, BCL-XL participates in the modulation of several important non-apoptotic cellular processes including the regulation of ER calcium stores, maintenance of the physiological conformation of VDAC, and promotion of the exchange of metabolites, including ADP, across the MOM [159,160,161]. BCL-XL can also interact with Beclin-1, thereby preventing stress-induced activation of autophagy [162]. Additionally, it participates in the maintenance of the homeostasis of T cells and the survival of hematopoietic stem cells [163,164]. Lastly, endogenous BCL-XL is critical for MYC-driven lymphomagenesis in mice [165].

### 5.2. BCL-XL and Hematologic Malignancies

*BCL2L1* chromosome region 20q11.21 coding for BCL-XL is amplified in diverse malignancies [136]. In analogy to MCL1-amplified tumors, targeted downregulation of BCL-XL in the tumors with 20q11.21 gains was associated with decreased survival or the induction of programmed cell death. Upregulated BCL-XL correlated with adverse outcome in AML [166]. Recently, BCL-XL has gained attention as one of the principal mediators of venetoclax resistance.

### 5.3. BCL-XL Inhibitors

Despite its well-established role in blocking the apoptotic cascade, specific inhibitors of BCL-XL have shown limited single-agent activity in the experimental therapy of hematologic malignancies [167]. The recent discovery of the essential role of BCL-XL in mediating resistance to venetoclax reestablishes the potential therapeutic role of BCL-XL inhibitors as venetoclax sensitizers [168]. The combination of venetoclax and navitoclax was well tolerated in patients with AML and lymphoblastic lymphomas [169]. Novel, dual BCL2 and BCL-XL inhibitors, e.g., AZD0466 or pelcitoclax (APG-1252), are currently under clinical development [170,171].

## 6. BCL-W and BFL-1/A1

### 6.1. BCL-W

In contrast to the three above-mentioned major anti-apoptotic BCL-2 family proteins, the remaining three, i.e., BCL-W, BFL-1/A1, and BCL-B have been much less studied. BCL-W was discovered in 1996 and was later also confirmed as an anti-apoptotic BCL-2 family protein similar to BCL-XL, blocking the pro-apoptotic potential of BAX and BAK as well as interacting with multiple members of the BH3-only group [156,172]. BCL-W is expressed in multiple tissues including the testes, colon, brain, and cells of hematopoietic origin. Mice with eliminated expression of BCL-W are viable, though males are infertile due to excessive loss of Sertolli and germ cells [173,174,175]. Furthermore, BCL-W was reported to contribute to the survival of epithelial cells in the gut and B lymphocytes [173,176]. However, increased expression of BCL-W is linked to tumorigenesis in various types of cancers [177]. Among hematologic malignancies, BCL-W is highly expressed in a subset of c-MYC-expressing DLBCL and Burkitt lymphomas as well as B-CLL cells, although it appears dispensable for their survival [176,178]. Thus far, no BCL-W-specific BH3 mimetics have been discovered, most likely due to its high conformational flexibility.

### 6.2. BFL-1/A1

Similarly to BCL-W, BFL-1 (human)/A1 (murine) was discovered in 1996 as yet another anti-apoptotic protein from the BCL-2 family. It contains three BH1-3 domains and a glutamine stretch in place of a putative BH4 domain [179,180]. Even though A1 is expressed in multiple mouse embryonic tissues, its genetic elimination causes only a minor defect in dendritic cell survival, while its shRNA-mediated downregulation in vivo impairs T cell differentiation and granulocyte survival [181,182]. Interestingly, A1 enhances BCL-2 and MCL-1 mediated survival of hematopoietic cells and its expression can be activated by inflammatory cytokines TNF-α and IL-1-β, likely in an NF-κB-dependent manner [183,184]. BFL-1/A1 is overexpressed in therapy-resistant lymphoma cell lines, and its expression enhances the survival of c-MYC-dependent B-cell lymphomas and EBV LMP1 protein-expressing Burkitt lymphoma [185,186,187,188]. Moreover, overexpression of BFL-1 enhances the resistance of c-MYC- and BCL2-expressing double-hit lymphomas to venetoclax, while its downregulation sensitizes them to apoptotic stimuli [189,190]. Simultaneous expression downregulation of two anti-apoptotic BCL2 proteins with short half-lives, BFL-1/A1 and MCL1, by CDK9 inhibitors has been shown to overcome the resistance of DLBCL cells to venetoclax [151,190]. These preclinical data provide a clear rationale for the potential of BFL-1/A1-targeting BH3 mimetics in the experimental therapy of cancer [191].

### 6.3. Disadvantages of BH3 Mimetics

BH3 mimetics belong to targeted anti-cancer agents. Consequently, their anti-tumor efficacy depends upon the expression of their respective targets, the anti-apoptotic BCL2 proteins. In translation, all BCL2-negative malignancies (e.g., Burkitt lymphoma, BCL2-negative DLBCL, or a substantial part of MM) are inherently “resistant” to BCL2 inhibition with venetoclax but still may be effectively targeted with other BH3 mimetics, e.g., MCL1 inhibitors. Importantly, for the full deployment of their pro-apoptotic activity, the anti-apoptotic BCL2 proteins must be primed for death, or “loaded” with pro-apoptotic BH3-only proteins, and the effectors of the apoptotic pathway must remain functional [4,192]. Leukemia or lymphoma cells with mutated *BAX*, or BCL2 protein not primed for death, will not respond to venetoclax. Currently, there are few reliable predictive markers of the sensitivity to venetoclax or other BH3 mimetics. Techniques such as (dynamic) BH3 profiling remain limited to a few highly specialized translational laboratories and cannot be considered a standard diagnostic procedure [193,194]. Lastly, the current standard of long-term dosing of venetoclax may be associated with cumulative toxic side effects. The long-term exposure of leukemia stem cells or lymphoma “persister cells” to venetoclax may lead to the development of the above-described adaptive changes and relapse of the resistant disease.

## 7. Conclusions

BH3 mimetics represent innovative molecules that have proved effective both individually and in combination with a diverse group of conventional, targeted, or biological anti-tumor agents in various types of hematologic malignancies. Their capability to induce BAX/BAK1-dependent apoptosis without requiring the activation of canonical DNA damage pathways enables the eradication of non-dividing malignant cells with complex karyotypic alterations, defects in *TP53*, and inactivation of other key tumor suppressor genes. Besides their well-described mechanistic pro-apoptotic activities, recent data have brought evidence of important non-canonical anti-tumor functions, namely modulation of oxidative phosphorylation and other vital mitochondrial functions. The frequent development of resistance to venetoclax monotherapy (and, preclinically, to other single-agent BH3 mimetics) calls for novel, more effective treatment combinations. Alternatively, BH3 mimetics might be used in sequential protocols for the targeted eradication of minimal residual disease following debulking with standard regimens. The currently running clinical trials that incorporate venetoclax and next-generation BH3 mimetics will soon shed light not only on the efficacy of these experimental regimens but also on other critical aspects associated with BH3 mimetic treatments, including safety issues, the optimal length of therapy, and reliable biomarkers of sensitivity or resistance.

## Figures and Tables

**Table 1:** Localization, non-apoptotic functions, and knockout phenotypes of anti-apoptotic BCL2 proteins.

Protein	Molecular Weight (kD)	Subcellular Localization	Non-Apoptotic Functions	Knockout Mice
BCL2	25	Mitochondria, ER, nuclear envelope	Autophagy, OxPhos, regulation of Ca^2+^	Viable (50% die after 1–6 weeks). Rapid loss of immune cells, increased sensitivity to apoptotic stimuli.
BCL-XL	30	Mitochondria, ER, cytosol	Shape of mitochonria, autophagy, DNA-damage response, OxPhos, regulation of Ca^2+^	Lethal around embryonic day 13: neuronal degradation, decreased survival of lymphocytes and erythrocytes, increased apoptosis of hematopoietic precursors in liver.
MCL1	40	Mitochondria, ER, nuclear envelope, cytosol	Shape of mitochonria, autophagy, OxPhos, regulation of Ca^2+^	Preimplantation lethality: reduction in B and T lymphocytes, block of lymphocyte differentiation.
BCL-W	19	Mitochondria, cytosol	Shape of mitochonria, autophagy	Viable. Male sterility and testicular degeneration.
A-1/BFL-1	20	Mitochondria, cytosol, nucleus	Autophagy	Viable. Increased neutrophile apoptosis.

ER = endoplasmic reticulum, OxPhos = oxidative phosphorylation.

**Table 2:** BCL2 expression and mechanisms of BCL2 deregulation in hematologic malignancies.

Disease	Dependence on BCL2 and/or MCL1	Proportion of BCL2 Positivity	Molecular Mechanism(s) of BCL2 Dysregulation	Proportion of Patients with the Aberration
FL	BCL2	>90%	*t(14;18)(q32;q21)*	90%
			*BCL2* mutations	
DLBCL-GCB	BCL2 and MCL1	70%	*t(14;18)(q32;q21)*	30%
			*BCL2* mutations	10%
			Hypermutation of *BCL2* promotor	
DLBCL-ABC	BCL2 and MCL1	90%	*18q21* amplifications	20%
			*t(14;18)(q32;q21)*	5%
MCL	BCL2 > MCL1	100%	*18q21* gain	20%
			Cyclin D1 overexpression	100%
MZL	BCL2	80%		
CLL	BCL2	100%	*13q14* loss (miR-15a, miR-16-1)	50%
BL	MCL1	0%		
MM	MCL1 > BCL2	60%	Cyclin D1 overexpression	20%
AML	BCL2 and MCL1	70–100%		

ABC = activated B-cell; AML = acute myeloid leukemia; BL = Burkitt lymphoma; CLL = chronic lymphocytic leukemia; DLBCL = diffuse large B-cell lymphoma; FL = follicular lymphoma; GCB = Germinal center B-cell; MM = multiple myeloma; MCL = mantle cell lymphoma; MZL = marginal zone lymphoma.

**Table 3:** Clinically approved indications of venetoclax.

Disease	Regimen	Treatment Status
CLL	Venetoclax monotherapy	In the presence of *TP53* aberration for patients, who have failed or who are not suitable for Bruton tyrosine-kinase inhibitor
		In the absence of *TP53* aberration for patients, who have failed both chemoimmunotherapy and Bruton tyrosine-kinase inhibitor
CLL	Venetoclax + Rituximab	Patients, who relapsed after at least one line of therapy
CLL	Venetoclax + Obinutuzumab	Untreated patients
AML	Venetoclax + hypomethylating agent	Newly dg. patients ineligible for intensive therapy

AML = acute myeloid leukemia, CLL = chronic leukocytic leukemia.

**Table 4:** Selected clinical trials that incorporate venetoclax in experimental therapy of hematologic malignancies.

Agent(s)	Mode of Action of the Agents Used in Combination with Venetoclax	Study Phase	Target Disease	Estimated Study Completion Date	ClinicalTrials.gov Identifier (Other Identifier)
Venetoclax + Acalabrutinib +/− Obinutuzumab	Bruton tyrosine-kinase inhibitor + anti-CD20 antibody	3	CLL	March 2024	NCT03836261
Venetoclax + Ibrutinib	Bruton tyrosine-kinase inhibitor	3	MCL	September 2022	NCT03112174 (SYMPATICO)
Venetoclax + Ibrutinib + Obinutuzumab	Bruton tyrosine-kinase inhibitor + anti-CD20 antibody	3	CLL	June 2027	NCT03737981
Venetoclax + pirtobrutinib + rituximab	Bruton tyrosine-kinase inhibitor + anti-CD20 antibody	3	CLL	January 2027	NCT04965493
Venetoclax + Ublituximab + Umbralisib	Anti-CD20 antibody + inhibitor of phosphatidylinositide 3-kinase	3	CLL	March 2025	NCT03801525
Venetoclax + Lenalidomide + Obinutuzumab	Immunomodulatory agent + anti-CD20 antibody	2	FL	November 2026	NCT03980171
Venetoclax + ivosidenib +/− azacitidine	Inhibitor of isocitrate dehydrogenase (IDH1) + hypomethylating agent	2	*IDH1-*Mutated HM	September 2021	NCT03471260
Venetoclax + R-CHOP	Standard immunochemotherapy R-CHOP	2	Richter syndrome	December 2025	NCT03054896
Venetoclax + Rituximab + Bendamustine	Anti-CD20 antibody, chemotherapy agent	2	MCL	March 2025	NCT03834688
Venetoclax + Carfilzomib and Dexamethasone	Proteasome inhibitor and corticosteroid	2	MM	August 2025	NCT02899052
Venetoclax + dasatinib	Tyrosine-kinase inhibitor	2	Early chronic phase CML	December 2040	NCT02689440
Venetoclax + Ponatinib + Decitabine	Tyrosine-kinase inhibitor + hypomethylating agent	2	Ph+ AML, blast-phase CML	September 2024	NCT04188405
Venetoclax + Low-dose Homoharringtonine + Azacitidine	MCL1 and BCL-XL targeting alkaloid + hypomethylating agent	2	AML	March 2024	NCT04824924
Venetoclax + Azacitidine + MBG453	Hypomethylating agent + TIM-3 immune check point inhibitor	2	AML	January 2026	NCT04150029 (STIMULUS-AML1)
Venetoclax + Ibrutinib	Bruton tyrosine-kinase inhibitor	2	WM	June 2029	NCT04273139
Venetoclax + Azacitidine + Pevonedistat	Hypomethylating agent + NEDD8-activating enzyme inhibitor	2	AML	March 2024	NCT04266795 (PEVENAZA)
Venetoclax + Polatuzumab Vedotin + Rituximab	Anti-CD79-MMAE antibody-drug conjugate + anti-CD20 antibody	2	MCL	December 2025	NCT04659044
Venetoclax + liposomal vincristine	Anti-mitotic agent	2	ALL	April 2021	NCT03504644
Venetoclax + R-ICE	Anti-CD20 rituximab + salvage immunochemotherapy R-ICE	2	DLBCL	October 2021	NCT03064867
Venetoclax + Eprenetapopt	Mutant *TP53* reactivator	2	AML	Juna 2023	NCT04419389

ALL = acute lymphoblastic leukemia; AML = acute myelogeneous leukemia; B-NHL = B cell non-Hodgkin lymphomas; R-CHOP = rituximab + cyclophosphamide + doxorubicin + vincristine + prednisone; CLL = chronic lymphocytic leukemia; CML = chronic myelogeneous leukemia; DLBCL = diffuse large B-cell lymphoma; EPOCH-R = etoposide, prednisone, vincristine, cyclophosphamide, doxorubicin, rituximab; FL = follicular lymphoma; HM = hematologic malignancies; MCL = mantle cell lymphoma; MM = multiple myeloma; R-ICE = Rituximab + ifosfamide + carboplatin + etoposide; WM = Waldeström macroglobulinemia.

**Table 5:** Selected clinical trials that incorporate next-generation BCL2 inhibitors in experimental therapy of hematologic malignancies.

Next-Generation BCL2 Inhibitors +/− Other Agents	Mode of Action of Agent(s) Used in Combination with the Next-Generation BCL2 Inhibitor	Study Phase	Disease Status	Estimated Study Completion Date	ClinicalTrials.gov Identifier (Other Identifier)
BGB-11417	Single-agent BCL2 inhibitor	1	mature B-cell malignancies	May 2024	NCT04883957
BGB-11417 +/− zanubrutinib	Bruton tyrosine-kinase inhibitor	1	mature B-cell malignancies	August 2023	NCT04277637
BGB-11417	Single-agent BCL2 inhibitor	1	Myeloid malginancies	February 2024	NCT04771130
BGB-11417 +/− Carfilzomib + Dexamethasone	Proteasome inhibitor + corticosteroid	1/2	MM	September 2025	NCT04973605
S65487 + Azacitidine	Hypomethylating agent	1/2	AML	March 2024	NCT04742101
S65487	Single-agent BCL2 inhibitor	1	AML, B-NHL, MM, CLL	August 2023	NCT03755154
S65487/VOB560 + MIK665/S64315	MCL1 inhibitor	1	HM	January 2025	NCT04702425
APG-2575	Single-agent BCL2 inhibitor	1	CLL/SLL	January 2022	NCT04215809
APG-2575 + Pomalidomide/Dexamethasone or Daratumumab + Lenalidomide + Dexamethasone	Immunomodulatory agents pomalidomide and lenalidomide, anti-CD38 antibody daratumumab, corticosteroid dexamethasone	1/2	MM	December 2023	NCT04942067
APG-2575 a Azacitidine	Hypomethylating agent	1	AML	October 2023	NCT04964518
FCN-338	Single-agent BCL2 inhibitor	1	CLL/SLL	June 2024	NCT04682808

AML = acute myelogeneous leukemia; B-NHL = B-cell non-Hodgkin lymphoma; CLL = chronic lymphocytic leukemia; HM = hematologic malignancies; MM = multiple myeloma; SLL = small lymphocytic lymphoma.

**Table 6:** Selected clinical trials that incorporate MCL1 inhibitors in experimental therapy of hematologic malignancies.

Agent(s)	Mode of Action of Agent(s) Used in Combination with the MCL1 Inhibitor	Study Phase	Disease Status	Estimated Study Completion Date	ClinicalTrials.gov Identifier (Other Identifier)
AZD5991 + Venetoclax	BCL2 inhibitor	2	AML	March 2023	NCT03218683
MIK665/S64315 + Azacytidine	Hypomethylating agent	1/2	AML	March 2024	NCT04629443
MIK665/S64315 + VOB560/S65487	BCL2 inhibitor	1b	lymphoma, MM, AML	January 2025	NCT04702425
MIK665/S64315	Single-agent MCL1 inhibitor	1	lymphoma, MM, AML	Completed	NCT02992483
PRT1419	Single-agent MCL1 inhibitor	1	lymphoma, MM, AML, MDS	September 2022	NCT04543305
AMG-176	Single-agent MCL1 inhibitor	1	MM, AML	December 2023	NCT02675452
MIK665/S64315 + Venetoclax	BCL2 inhibitor	1	AML	January 2024	NCT03672695
ABBV-467	Single-agent MCL1 inhibitor	1	MM, AML	Terminated	NCT04178902
AMG-176 + Venetoclax	BCL2 inhibitor	1	R/R HM	Suspended (Safety)	NCT03797261

AML = acute myelogeneous leukemia; HM = hematologic malignancies; MDS = myelodysplastic syndromes; MM = multiple myeloma.

## Abbreviations

ABC	Activated B-Cell
AL (amyloidosis)	Amyloid Light (chain)
AML	Acute Myeloid Leukemia
BCL2	B-cell Lymphoma 2
BH3	BCL2 Homology 3
BTK	Bruton’s Tyrosine Kinase
CDK	Cyclin-Dependent Kinase
CLL	Chronic Lymphocytic Leukemia
DLBCL	Diffuse Large B-cell Lymphoma
ER	Endoplasmic Reticulum
GCB	Germinal Center B-cell
HM	Hematologic Malignancies
HMA	Hypomethylating Agents
MCL	Mantle Cell Lymphoma
MCL1	Myeloid Cell Leukemia 1
MDM2	Mouse Double Minute 2 Homologue
MM	Multiple Myeloma
MOM	Mitochondria Outer Membrane
OxPhos	Oxidative Phosphorylation
TM	Transmembrane Domain
RCD	Regulated Cell Death
R-CHOP	Rituximab + Cyclophosphamide + Hydroxydaunomycin + Oncovin + Prednisone
ROS	Reactive Oxygen Species
R/R	Relapsed/Refractory
